# Wearing face masks impairs dyadic micro-activities in nonverbal social encounter: A mixed-methods first-person study on the sense of I and Thou

**DOI:** 10.3389/fpsyg.2022.983652

**Published:** 2022-12-15

**Authors:** Johannes Wagemann, Christian Tewes, Jonas Raggatz

**Affiliations:** Institute for Waldorf Education, Inclusion and Interculturalism in Mannheim, Alanus University of Arts and Social Sciences, Alfter, Germany

**Keywords:** nonverbal social interaction, first-person perspective, mental micro-activities, direct social perception, I-Thou sense, weak vs. strong embodiment

## Abstract

The COVID-19 pandemic has manifold negative consequences for people around the world, of which the psychosocial ones have been rather underrepresented in the public eye. Regarding social distancing measures, there is already some experimental work demonstrating that the use of face masks has detrimental effects on various aspects of social cognition such as emotion reading, face identification, and perceived closeness of persons. However, while these findings provide important clues, they do not shed light on what people experience when interacting in real life in a masked society. Therefore, in critical distance to cognitivist accounts and taking Direct Social Perception (DSP) approaches seriously, we developed a first-person experimental design and conducted a study with thirty-four participants in a dyadic setting with two conditions (without vs. with face mask). Data were analyzed with mixed methods including in-depth qualitative coding at three levels, code relations analyses, and various statistical tests. Results yielded significant differences across conditions at all qualitative levels, comprising, for example, expressive behavior, and, in particular, significant decreases of content-independent, complimentary mental micro-activities. In the context of DSP, we argue in the paper that these activities suggest the constitution of a quasi-sensory modality – conceived as I-Thou sense – that oscillates between strongly and weakly embodied mental activities, as the analyses show. In sum, this study suggests that mask-wearing impairs both functional directions of mental activity in relation to more or less embodied experience and thus intervenes deeply in fundamental processes of social perception and interaction.

## Introduction

Many governments around the world have mandated the wearing of face covering masks in response to the global COVID-19 pandemic to prevent the spread of the virus. While the medical benefits of face masks are mostly undisputed (Brooks and Butler, 2021; Howard et al., 2021; for review, see Spitzer, 2020), little is known about their (social) psychological consequences. What is known so far is that face masks impair social cognition by hiding areas of the face that carry cues to emotions, identity, speech information, and other social aspects such as trustworthiness, attractiveness, and age (Carbon, 2020; Freud et al., 2020; Biermann et al., 2021; Marini et al., 2021; Noyes et al., 2021). For instance, recent studies found that emotional recognition was made significantly more difficult by the presence of a mask (Carbon, 2020; Gori et al., 2021; Grahlow et al., 2022; Grenville and Dwyer, 2022) or that face masks disrupt holistic processing abilities and thus have impact on facial identification and social cognition (Freud et al., 2020; Marini et al., 2021; Jeevan et al., 2022; Stajduhar et al., 2022; Thorley et al., 2022). Furthermore, face masks make target persons appear less close (Grundmann et al., 2021; Kastendieck et al., 2022), reduce interpersonal trust (Malik et al., 2021; Marini et al., 2021), and impact the recall of spoken sentences and voice radiation in general (Pörschmann et al., 2020; Homans and Vroegop, 2021; Truong et al., 2021). At the same time, there is also some evidence that face masks increase facial attractiveness (Hies and Lewis, 2022; Parada-Fernández et al., 2022; Pazhoohi and Kingstone, 2022).

However, while these findings already stake out the scope of psychological phenomena affected by mask use, they are limited in terms of what people experience in real-life social interactions and how those interactions actually change as a result. Firstly, this is because all the cited studies deployed facial stimuli that were created by using image processing software or images of people wearing a mask. Although such common approaches allow for a high degree of experimental control and large sample sizes due to automated procedures in online survey designs, they circumvent the study of psychological effects of face masks on a more natural, holistic, and dynamic level. Secondly, it is questionable whether standardized data collection *via* measurement of known constructs is sufficient for the purposes of the experiments, or whether lived experience and mental agency should be accessed in a more direct way. Since the latter may uncover unexpected and more elusive aspects of social cognition and interaction (Wagemann and Weger, 2021), the current study was designed to investigate the impact of face masks in an almost natural setting relying mostly on qualitative self-report data without, however, neglecting the virtues of quantitative analysis.

### From Descartes to theory theory and simulation theory

In theoretical terms, a mixed-methods first-person study invites us to more precisely explore from a conceptual and empirical perspective philosophical approaches to social cognition and interaction, which have undergone a significant transformation in recent decades. In the cognitive sciences the traditional answer to the problem of other minds – which has its roots in Cartesian substance dualism – claims that certain internal mechanisms explain how we gain access to the mental (e.g., emotional) states of others and understand their intentions. There is, in Cartesian terms, no direct perception of the world – including the perception of other minds. This is so because the *res cogitans* has to *interpret* the signs of the nervous system (in the spinal gland) that transmit the received information of the sense organs. It is this idea, namely that we do not have direct contact with the external world, that is the starting point of almost every contemporary theory of social perception. Thus, one of the leading theories of social cognition, the theory theory (TT) states, roughly speaking, that we acquire knowledge about other people’s mental states by construing a theory based on our own perceptual experience and inference (Bretherton and Beeghly, 1982; Schaafsma et al., 2014). TT claims that we connect sensory data with mental states and behavioral states (input/output states) by assuming causal connections between them, which can be refined, for instance, by exploring these connections in more detail (Shanton and Goldman, 2010). For understanding others’ intentions, we then ascribe folk psychological categories such as beliefs and desires to other people by causal inferences. Another well-known attempt to explain our mind-reading capabilities is the so-called simulation theory (ST). In contrast to TT, the simulation theory rejects the idea of theoretical inferences as the basic mechanism for understanding other’s intentions and for predicting their prospective actions. Early predecessors of ST – such as the hermeneutical and aesthetic approach to other persons and to artwork – assume that feeling with others (“Einfühlung”) or reexperiencing (“Nacherleben”) their mental life are the key aspects for intersubjective understanding (Dilthey, 1927, p. 47; Lipps, 1903). In a similar vein, contemporary proponents of ST hold the view that with our imaginative powers we put ourselves in the shoes of others to grasp what people feel or think in different social circumstances (Gordon, 1986; Heal, 1986; Shanton and Goldman, 2010). As Goldman puts it, a “mentalizer” simulates the mental situation of another person by pretending states in herself so as to comprehend what she might presently be feeling and thinking and how she might act in the future (Goldman, 2006, p. 258). There is, however, another variant of simulation theory that is not directly concerned with the ascription of propositional attitudes but with the experience of the basic motor intentions of other people in the context of sensory-motor interactions. In contrast to the simulation theory, which ascribes mental activities at the personal level of descriptions, this complementary variant builds on the mirror neuron system found in monkeys (Gallese et al., 2004) and humans (Freedberg and Gallese, 2007). According to Gallese (2003), these empirical findings imply that the correlated brain activities simulate forthcoming events for predicting social interactions. He calls this process “embodied simulation” and characterizes it as an automatic and unconscious mechanism, which he conceives as the functional basis of empathy and social cognition (Gallese, 2003, p. 524; Freedberg and Gallese, 2007).

### Direct social perception and interpersonal sensitivity

Nevertheless, the notion of indirect social cognition underlying these two and other variants of the theory of mind has been challenged by embodied and phenomenologically inspired approaches. Drawing on the work of Scheler (1954), Merleau-Ponty (1964), Gallagher (2008), Stein (2008) and others have in recent years delineated a theory of direct perception from the second-person perspective, which rejects the idea that mental states are hidden and unobservable entities. On the contrary, we perceive other people’s emotions or intentions immediately, for instance, by voice intonation, facial expressions, gestures, or entire body postures (Gallagher, 2008; Reddy, 2008). One core idea in support of this view holds that *expressive embodied behavior* is a constitutive part of mental states. When we observe such states, we are perceiving what the other is experiencing from the first-person perspective (Krueger and Overgaard, 2012).

Furthermore, the observation of expressive behavior is simultaneously intertwined with the encounter of another subject. When we see another person with an expression of sadness or grief in her face or we observe her entire body posture, we are confronted not only with a mental state of another person but are encountering the other as the subject of those mental states. Matthew Ratcliffe, in particular, has explored this distinction of social content and interactants and the relationship between the two in more detail. Referring to Buber’s (1958) famous I and Thou relation (1958), Ratcliffe argues that we are not facing a “Thou” as an entity that we “recognize and address then as Thou” (Ratcliffe, 2007, p. 154), but rather the I-Thou relation is a stance – or a sense which encloses a special field of experience – that already requires a specific activity and openness on the part of the interactive participants to the other as a Thou (Ratcliffe, 2007). A similar notion of “interpersonal sensitivity,” which goes beyond emotional empathy, has been studied empirically. In this context, interpersonal sensitivity is understood as “accuracy in judging the meanings of cues given off by expressors, as well as accuracy in noticing or recalling cues” (Hall et al., 2005, p. 237). What supports Buber’s and Ratcliffe’s concept of dialogic interaction is that there is a positive correlation between participants’ own interpersonal sensitivity and the accuracy of their assessment of their partner’s interpersonal sensitivity (Carney and Harrigan, 2003). In our context, however, one limitation of this approach is that, by measuring *via* standardized survey instruments, it presupposes the specific content of cues to be observed by the participants and thus is unable to capture novel structural and dynamic dimensions of the interactants’ encounters. Another limitation is that, in recent psychopathological research, interpersonal sensitivity is mostly associated with negative personality traits such as low self-esteem and negative self-image that result in high alertness to the expectations of others (Meisel et al., 2018; Lei et al., 2021), which seems to neglect the general functional role of disclosing interpersonal sensitivity in social interaction.

### Extending DSP by (potentially) conscious attention dynamics

In terms of direct social perception (DSP), the gaze plays a proactive and receptive dual role in social encounters, as already noted by Simmel (1921) and Sartre (1956). Apart from Sartre’s (one-sided) notion that the gaze of the other is constitutive of myself as an objectified person, his analysis of the phenomenon of shame suggests that it involves a responsiveness to the gaze of the other that can be described as the permanent possibility of “being-seen by another” (Sartre, 1956). From this we can infer an important distinction between two types of experiences, namely to be passively seen-by-another and to actively look-at-another, which both involve the specific personal I-Thou stance. This dual function of eye gaze has also been empirically explored since the 1960s, in relation to various phenomena such as intimacy and dominance (Argyle and Cook, 1976; Ellyson et al., 1981; Jarick and Kingstone, 2015) or turn-taking behavior during conversation (Kendon, 1967; Ho et al., 2015). In the latter, looking at the other and being looked at by the other take place in that speakers change between averting their gaze and turning it toward the listener, while listeners gazed at speakers most of the time (Kendon, 1967). Conversely, listeners tend to avert their gaze and increase overt behavior such as head shifts or gesturing before starting to speak (Harrigan, 1985). However, at least since Posner’s influential research, it has been clear that there are also covert attention dynamics which cannot be captured by measurement of overt or external behavior but need to be considered in their own right too (Posner, 1988; Posner and Petersen, 1990), for example, when a detective pretends to read the newspaper while observing people in the periphery of her visual field. While it is often assumed that both overt and covert forms of attention in turn-taking are due to implicit, i.e., unconscious brain processes (e.g., Sato et al., 2016; Bögels, 2020), we have suggested that eye movements are a visible metaphor for more subtle processes of conscious attention regulation that are quite accessible to first-person observation (Wagemann and Weger, 2021). In this regard, we distinguish between two mental modes of listening, namely, first following the other person’s utterances with undivided attention, and then, while continuing to listen, detaching one’s attention to some extent from the other and developing one’s own impulses to (re-)act, e.g., to say something back. Notably, the change from the first (“devotion”) mode to the second (“self-assertive”) mode can (though need not) be accompanied by overt behavior (Corps et al., 2018). To capture the more subtle attentional dynamics that oscillate between one’s own and the other’s domain and which could be more fundamental to phenomenal experience in social interaction than their overt or neural reverberation, we need to pursue a first-person research paradigm and develop an appropriate theoretical approach.

A notion that comes closest to answering our question of not only if but how direct social perception might be possible can be found in Rudolf Steiner’s idea of the sense of I and Thou (Steiner, 1964), which he, like Buber, developed in the 1910s. This is because Steiner’s first-person phenomenological approach focuses not on specific mental content (to be decoded in terms of social functions or communication purposes) but instead examines underlying mental processes from a structural perspective. Unlike Sartre, however, he explores in a balanced way the mutually alternating roles of proactively sending and receptively opening mental activity, which ultimately result in the “other self” being perceived as a mental agent on an equal footing and with her own inimitable individuality. In the current study, our more concrete question is directed at whether this view, initially supported in a previous study (Wagemann and Weger, 2021), can be further strengthened by confronting it with an experimental setting in the form of social distancing measures. Here, we are concerned with the transition from nonverbal social interaction without mask to that with mask, in order to use this independent variable as a potential indicator of altered experience and mental agency in dyadic encounters. Additionally, we decided to enable participants to focus more thoroughly on hitherto unnoticed subtle inter-mental and intra-mental activities without the distraction of complex simultaneous symbolic (verbal) communication processes. Furthermore, our study aims at contributing to the question of how the presence of the mask in social interactions during the Corona crisis might affect the ability of direct perception and interaction in the social realm.

### Hypotheses

In analyzing the data, we will proceed step by step, examining the data at different levels, progressing from more general aspects to focusing on mental or attentional micro-activities (e.g., Wagemann, 2022). For these, we formulate the following two hypotheses: (1) Mental activity in (nonverbal) social interaction appears in two typical forms which participants can introspectively observe and report (proactive-focusing, receptive-opening) and which are partly expressed *via* overt or bodily behavior and partly act independently. (2) In the transition from interaction without mask to interaction with mask, these forms of mental activity change in the frequency of their occurrence and their characteristic composition. More specifically, we hypothesize a decrease or impairment of these micro-activities in the transition from the first to the second condition, which is in line with the findings presented above (Carbon, 2020; Gori et al., 2021), but which also has the potential to provide a deeper understanding of these effects. If these hypotheses can be empirically strengthened, this could have important implications both for a general, psychological-philosophical conception of social interaction in terms of DSP and for the often insufficiently considered psychological and cultural implications of health policy measures.

## Experimental procedure

The methodological starting point of our investigation is the irreducible phenomenal first-person perspective of experience. This does not exclude, however, the important contributions of neuroscientific or other behavioral research to the externally measurable underpinnings of mental life. Nevertheless, we maintain that operational definitions and functional specifications of mental processes are frequently in danger of losing sight of the phenomenality of conscious experience and subjectivity (Weger and Wagemann, 2015; Kotchoubey et al., 2016). Actually, phenomenal consciousness in its structural coherence provides functional dimensions and empirical evidence which are often neglected in consciousness research (Hatfield, 2005; Locke, 2009; Cleeremans and Tallon-Baudry, 2022). Consequently, a methodologically justified research procedure is needed to deal with conscious phenomena *in themselves*. Only after having established a rigorous phenomenal description and categorization of conscious experience can one start to explore how a non-reductive but integrative mind science from the first-, second- and third-person perspective is to be accomplished (Kotchoubey et al., 2016).

Thus, taking experience from the first-person perspective seriously, we share the conviction with contemporary phenomenological approaches that the content and acts of conscious processes need a qualitative research procedure which aims at specifying their invariant structures and dynamics of experience. Such a phenomenological approach, however, does not exclude the *quantitative* investigation of the phenomena being analyzed, such as different forms of social interaction (Creswell, 2009). Phenomenologically inspired mixed-method approaches focus on the *integration* of qualitative and quantitative (statistical) research procedures for a multi-perspective disclosure of the structures and dynamics in question (Martiny et al., 2021). This, in turn, opens up new research fields for neurophenomenological research projects (Tewes, 2018), indicating the possible integration of different research perspectives in the mind sciences, as highlighted above.

Against this background, we need to connect the question of mental agents’ micro-activities and direct social perception with the requirements and quality criteria of empirical research. This means developing an appropriate experimental design and task, capturing people’s immediate experience as directly as possible, analyzing the qualitative data in a reliable way that is balanced between inductive (bottom-up or data-driven) and deductive (top-down or theory-driven) perspectives, quantifying the qualitative results, and identifying purely quantitative aspects of the text data before subjecting both to statistical analyses. Here, we would like to note that in this triangulation of qualitative, qualitative-quantitative, and purely quantitative aspects of the data lies a unique selling point of the present study, allowing it to bridge the gap that often exists between real-life interaction and analytical standards. Against this background, the individual steps of the experimental procedure are explained below.

Firstly, our decision for a within-subjects design requires justification. One advantage of a within-subjects design is to ensure maximum control of the participants’ extraneous variables, which reduces noise and leads in many cases to greater statistical power (Charness et al., 2012). Another argument for within-subjects designs is that they are closer to real-life situations where the same person is exposed to a variety of demands. However, care must be taken to avoid unintended carry-over effects that may result from the specific sequence of the experimental conditions; this is usually accomplished by randomization (e.g., Carbon, 2020). The situation is different if, as in our case, a condition is intentionally placed on subjects that they have a defined reference or “baseline” when doing the task under the subsequent conditions. In a sense, this can be understood as a pretest-posttest design, where data is collected before and after the administration of a particular treatment. It must then be conceded, however, that the scope of possible generalization of the findings is limited to exactly this sequence of conditions and cannot simply be transferred to others. Taking these considerations into account, the question of what happens in the transition from an unmasked society to a society that is largely masked in public life can be investigated by limiting the procedure to the change from interaction without to interaction with masks. Another reason for this constraint stems from the mixed-methods approach of our study, which is not only concerned with quantitative results, but also with the qualitative phenomenality of participants’ first-person experience and activity. For this reason, qualitative data is collected and analyzed first before it is fed into various quantitative analyses, which requires additional research resources and means a shifted methodological focus compared to purely quantitative studies. Nevertheless, in order to complement and continue the current study, further investigations will be needed.

### Task and participants

The experiment was conducted in June 2020 at Alanus University (Campus Mannheim) as part of a course on Social Aesthetics for students of our B.A. program in Curative Education. The students were instructed by email and carried out the experiment independently at home, for which they received partial course credit. Various theories of social philosophy and psychology were covered in class before the experiment, but of course the study was not discussed before or during data collection and participants were not informed about the hypotheses. The participants were instructed to sit in a quiet room without external disturbances facing a partner at a distance of 1.5 m and to maintain eye contact for 5 min. Other forms of nonverbal communication (facial or other physical gestures, sign language, etc.) were explicitly forbidden. Additionally, participants were asked to observe as closely as possible what they did and experienced mentally, to pay attention to active and passive aspects of this as well as to their thinking, feeling, willing, and perceiving. Immediately after the first phase, participants were to note down their observations, pause briefly, if necessary, but not talk to their partner. For the second round, they were instructed to repeat the same procedure with face mask on and again immediately note down their observations afterwards (without talking to each other). Finally, the reports were sent in by email within 1 week.

Methodologically this setting is a compromise between, on the one hand, highly controlled laboratory conditions (but which often seem sterile and unrealistic) and, on the other, purely qualitative field work (where it is often difficult to move beyond ideographic characterizations to nomothetic regularities). The real-life conditions also include the participants’ independent implementation of the trial within the timescale of 1 week, its integration into their daily routine and the inclusion of known people as interaction partners.

A possible objection to this procedure could be that participants had up to a week to reflect on the task before performing it, which could have influenced the way they experienced themselves and their partners in the experimental situation. In particular, the instructions on how to perform the task without vs. with mask could have made it completely transparent to participants what the researchers were interested in, which thus risks expectancy bias. However, especially for first-person studies which focus on agentive phenomena, it is inevitable that the external conditions of the experiment are completely transparent to participants: this is necessary for them to comply with the given task correctly (unlike many social psychological studies in which participants are intentionally misled about the task content). Moreover, even with prior reflection, the pre-reflective forms of experience and mental activity targeted by the hypotheses are performed and become conscious only during the task, and thus remain unaffected by these constraints. The task was designed according to the occasion of this study and the related restrictions for experimental research during the first phase of the COVID-19 pandemic in Germany. The initial idea was to conduct a follow-up experiment closer to that of Wagemann and Weger (2021), but we were unable to work with large groups moving through the room and therefore opted for a dyadic and static setting (for more detailed information on the previous study, see the last part of see section “Results”). Furthermore, there were no face-to-face classes at the university during this time, only online courses, which explains why students could only work at home. The following two reasons supported limiting the task to nonverbal interaction *via* eye contact: firstly, the potential effect of the mask should not be relativized by either additional task content or verbal communication, as explained in the introduction; secondly, participants should be exposed to a somewhat unfamiliar situation that remains unchanged for several minutes in order to temporarily leave their daily routine and engage in more subtle observations.

Thirty-four persons (30 females, 4 males) aged between 21 and 50 years (*M* = 25.8) participated in the experiment. As with the experimental setting, we can also speak of a compromise between qualitative and quantitative criteria regarding sample size. For thematic saturation in qualitative in-depth studies, 20–30 participants are recommended by Dworkin (2012), but other authors also consider sample sizes below 20 to be appropriate (Francis et al., 2010; Guest et al., 2020). On the other hand, for nominal variables, a statistical power analysis aimed at chi-square tests with a medium effect size of *w* = 0.3 (Cohen, 1988) yields with β/α = 4 and a total sample size of 68 (34 subjects under two conditions) a test power (1-β) = 0.74 (Faul et al., 2007), which is slightly below a widely recommended power of 0.8. For metrical variables, a power analysis aimed at dependent-samples t-tests with corresponding parameters and a medium effect size of *d* = 0.5 yields a test power of 0.85. In summary, we consider the given sample size to be appropriate in the context of this mixed-methods study.

### Data acquisition

For the setting described above we applied the method of retrospectively written, open-ended self-reports, since it has several advantages over both other first-person and more standardized methods. This is not to say that we consider this to be the only option; rather it is the best compromise for this type of investigation, as will be explained briefly.

Firstly, unlike interview techniques (e.g., Vermersch, 1999; Petitmengin, 2006), written self-reports are non-reactive in that they are not elicited or triggered by specific questions, thus avoiding any influence by an interviewer. This is especially important for hypothesis-driven research, to prevent forms of the experimenter expectancy effect or related biases. Although these problems may not necessarily arise with trained interviewers, they would require additional safeguards and justifications, which we bypass here.

Secondly, the hardly avoidable time gap between participants’ immediate experience and verbalization is minimized, as interviews cannot be conducted immediately after the experiment due to the real-life setting. An even greater temporal proximity of data collection to mental processes could have been achieved with a think-aloud technique (Ericsson and Simon, 1993), but this would disturb the nonverbal dyadic interaction and therefore had to be ruled out. If the notes are jotted down immediately after each trial, the limit of 30 s given by Ericsson (2006) for reliable retrospective self-reports should not be significantly exceeded, although this obviously does not apply to the earlier stages of the task. However, it can be assumed that real-life social encounters, in contrast to abstract cognitive tasks, are accompanied by salient emotions, ensuring that the corresponding experiences and activities are remembered more clearly, more accurately, and over a longer period of time (e.g., Tyng et al., 2017).

Thirdly, especially with regard to the social interaction task, it seems obvious to prefer written self-reports over interviews, since the latter would virtually be a repetition of the dyadic experiment with another person (and would most likely involve different social dynamics) and thus could have backward biasing effects on the actual experiences of interest. In the context of a mixed-methods study, there is also the pragmatic advantage of a significantly smaller data volume for written self-reports, which reduces the analysis effort.

Compared to standardized questionnaire instruments, open-ended written self-reports are advantageous here because they do not carry the risk of cognitive bias due to implicit information about the research hypotheses in the items, which is particularly important when dealing with phenomena and their structural components not previously described by known constructs. In this vein, the possibility of obtaining phenomenologically thick or rich descriptions (e.g., Masrour, 2011; Byrne and Siegel, 2017) and discovering new aspects in the data that go beyond the previous theoretical framework can also be mentioned here as crucial benefits compared to standard survey accounts (for general discussion of first-person methodology, see Tewes, 2007; Weger and Wagemann, 2015). Of course, space allowing, a more in-depth discussion of first-person data collection methods, ranging from more standardized to more (micro-) phenomenological procedures, would be needed here. Nevertheless, we have hopefully made clear the justification for the data collection method used in this study.

### Data analysis

A major part of the analytical work followed this sequence: qualitative multi-level coding of the text data (section “Qualitative analysis”), qualitative and quantitative code relations analysis (section “Code relations analysis”), descriptive and inferential statistical analysis of code frequencies and other quantitative aspects of the protocols (see “Statistical analysis”). Thus, from a mixed-methods perspective, an approach is taken in which qualitative and quantitative data are collected concurrently, as they are different aspects of verbalized experience, but analyzed in sequential steps before being integrated in terms of the research question (Creswell, 2009).

#### Qualitative analysis

To begin with the purely qualitative stage, we decided to balance data-driven and theory-driven aspects by coding the self-reports on three levels with different thematic focuses (Corbin and Strauss, 1990). Accordingly, different methods were pursued on each level regarding the coding procedure and intercoder reliability testing, as explained in more detail below. In advance, complete sentences were determined as the largest and partial sentences down to one-word statements as the smallest data segments to be coded.

Regarding the first level, our intention was to explore the data with as simple a categorization as possible, while allowing as much data as possible to be covered. Initial explorations into the data suggested a dichotomous distinction, as participants’ experience refers either to currently observed aspects of the interaction between the partners or to their own, somewhat detached thoughts and emotional or bodily states. Starting from this rough distinction between “dyadic” and “monadic” aspects, we elaborated it in three sessions in which individual coding attempts by the authors were compared, discussed, and refined according to successively revised code descriptions. This process resulted in the following categories for Level 1:

1. Dyadic. This includes all formulations that have a clear reference to the other person or the interaction event, but also those that contain the mere attempt to build up a relationship or establish contact as well as the failure of such an attempt, as well as the withdrawal from a dyadic contact and even the avoidance of a dyadic external reference by turning back to oneself. In all these cases, the reference to the interaction partner is present, whether explicitly or implicitly. Thoughts that refer directly to the partner are also part of this category.
2. Monadic. This covers all statements that refer only to the participant’s own mental or physical state without explicit reference to the other person, statements that could also be made in other situations or before or after the experiment, speculations about the experiment (in general as well as regarding specific observations that do not refer directly to the other person), attempts to explain or justify the experience, observations that refer to accompanying aspects (e.g., time experience, spatial constellation of the persons).

While this procedure led to conceptually consistent code descriptions and 97% coverage of the data (764 codings, no intralevel overlap), it did not provide any quantitative measure of intercoder agreement. Hence, intercoder reliability was tested with one independent coder who was not previously involved in the project. For 100 randomly selected and re-coded segments, computation of Cohen’s Kappa yielded κ = 0.856, which can be seen as strong (McHugh, 2012) or almost perfect agreement (Landis and Koch, 1977).

On the second level, reported experiences were to be differentiated as precisely as possible, aiming at a thematic categorization as complete as possible while being manageable in terms of the number of categories. In addition, it should be mentioned that the focus here was primarily on more obvious aspects of first-person experience, which again is likely to capture a large part of the data. Here we changed the procedure in that we initially agreed on six major categories, which were then further subdivided and elaborated by one of the authors (JW), who then also did the coding of the complete data. In Table 1, the resulting twelve (sub-) categories are summarized and explained. This resulted in 89% coding of the whole data with 764 encoded segments and 872 codings, which indicates 14.1% (108 codings, distributed over 53 segments) of intralevel overlap (double or triple codings of same segments). About one quarter of the encoded data (189 segments) was randomly selected for intercoder reliability testing and independently coded by the other two authors. For these two sets of codings, computation of Cohen’s Kappa yielded a mean value κ_M_ = 0.699, which already corresponds to substantial (Landis and Koch, 1977) or moderate agreement (McHugh, 2012). Here, as suggested by several scholars, we conducted one feedback round to clarify understanding and interpretation of the categories regarding the inconsistencies (Campbell et al., 2013; O’Connor and Joffe, 2020), which resolved most of them and ultimately resulted in strong agreement (κ_M_ = 0.883). The adjustments required on this basis were included in the final coding at Level 2.

**Table 1:** Second coding level.

Level 2 – Multiple aspects		Description	Examples
1. Thoughts, reflections, memories		Situation-specific or more general thoughts, reflections, associations, speculations, and upcoming memories during the trial, possibly related to the content of the other categories	“I started thinking about the task and also about my own reactions” (P 1) “…and wonder if he perceives something like that in my gaze as well” (P 19) “Incompleteness characterizes the encounter” (P 23)
2. Feelings		Whole spectrum of one’s own positive, negative, or absent/neutral feelings (except bodily induced states), and temporal experience	
	2.1 Affective feelings	Basic affective feelings, moods, and states; social emotions and emotional attitudes toward the other person; change of feelings	“…I also feel a little depressed in the process” (P 2) “…and felt very close to her” (P 18)
	2.2 Metacognitive feelings	Execution of the task, i.e., related cognitive activities, succeeds easily, difficulty, or fails, possibly accompanied by corresponding feelings (e.g., comfort, effort, frustration)	“At the beginning, I found it very difficult to concentrate” (P 5) “Mental: overall tense process” (P 24) “The experiment went much easier and I was able to concentrate better on maintaining eye contact without wandering off” (P 27)
	2.3 Sense of time	Time seems to be stretched, or to pass quickly, e.g., in comparison of the experimental conditions	“…In the end, the time was up quickly and could have gone longer” (P 4) “It felt like an eternity” (P 26)
3. Body		One’s own bodily sensations and reactions	
	3.1 Sensation	Felt energy level, arousal/tension/relaxation indicated by increased heartbeat or breath; sensation of posture, etc.	“Breathing was difficult, and I felt the heat under the mask” (P 15) “…initially tense and in the course very relaxing, the exercise has a very decelerating effect and does lasting good” (P 24)
	3.2 Reaction	Externally observable: Blinking, watering eyes, yawning, etc. Smiling/laughing is included if it occurs unintentionally as a (automatic) impulse and without explicit interaction context	“…had to laugh frequently. My posture also changed: first it was rigid and somewhat tense; later it became looser and more relaxed…” (P 5) “I also noticed that I hardly had to blink at all” (P 25)
4. Observation		Physical appearance of the other person and attention regulation	
	4.1 Content / quality	Physical appearance of the interaction partner, e.g., eye color, posture, face and facial movements/expressions, emotions, becoming aware of the other; *perceptual content* appears clear/blurry/altered	“Over and over again I looked at the whole face” (P 4) “Over time, the counterpart became more and more blurred” (P 6) “You seem a little sad to me” (P 14)
	4.2 Attention regulation	Direction and scope of focus; changing between focused/defosued attention; clear/blurry/altered *view* (without specific content); concentration/distraction; distanced/immersed observation	“…as both participants were distracted by the unfamiliar situation” (P 6) “…this focus was primarily on the right eye of my counterpart from my perspective” (P 11) “The view of the other person became more global” (P 13)
5. Intentions		One’s own urges, wishes, and intentions, e.g., trying to concentrate, to escape, etc., referring to individual interactions or to the entire trial; not (yet) initiated or completed forms of action, or retrospective explanation of the purpose of an action; often related to Categories 5.2 and 6.1	“…I tried to focus on what I could see in my counterpart” (P 1) “… in order to make me aware of how the person is doing” (P 17) “Wanted to impulsively rip off her mask at the beginning of the silence” (P20)
6. Behavior and interaction		All forms of one’s own and the partner’s overt behavior during the dyadic interaction	
	6.1 Eye gaze	Mutual gazing, sending and receiving cues; dynamics and phenomenal quality of view	“…my eyes [were] just drawn to my partner’s eyes like a magnet” (P 18) “…because I have the feeling to communicate through the eyes” (P 19)
	6.2 Mirrored behavior	Contagious smiling/laughing, yawning, tension/relaxation, mirrored posture, breath, emotions	“I have to start laughing because the other person is laughing” (P 2) “…mirroring the other person, such as head posture or even the rhythm of breathing…” (VP 5)
	6.3 Other behavior	One’s own and the other’s behavior beyond 6.1 and 6.2 but also in dyadic or communicative context; one’s own prevented behavior (e.g., impulse control)	“…the height of the mask, which is corrected once by me” (P 3) “I had to suppress my laughter all the time” (P 10) “I could see the smile, the smirk and the attempt to remain serious…” (VP 16)

Coding categories with subcategories, descriptions, and exemplary excerpts from the data.

At the third Level, the procedure was rather theory-driven in that most of the categories were adopted from a previous study with a comparable task (Wagemann and Weger, 2021). For this reason, as agreed, two of the authors were not involved in the formulation of the categories and instead this was carried out by one author (JW) alone. However, initial coding attempts with the adopted categories revealed the need to adapt them according to the modified task and to introduce additional categories (Table 2). Besides the four main categories representing the two mental micro-activities of a proactive-focused (PF) and a receptive-opening (RO) gesture observed as emanating from Person A or B each (main categories 1 to 4, see above hypothesis 1), two more holistic and two more specific categories were added. This is because there were many places in the protocols where individual micro-activities were not reported, but whether or not a dynamically integrated or resonant connection of the partners was perceived (categories 5.1 and 5.2). In addition, explicit negations of PF-B and RO-A activity occurred under the mask condition, which can be characterized more precisely as protection from being looked at by the other person (cat. 2.1) and as inhibition of the possibility of opening up and giving space to what comes from the other person (cat. 3.1). Since all these categories comprise more subtle aspects of first-person experience or activity, it is not surprising that a significantly smaller portion of 23% of the data could be coded at this level, resulting in 198 encoded segments, 201 codings, and an intralevel overlap of 5% (three double encodings). Intercoder reliability was tested in the same way as at Level 2, but with half of the encoded segments yielding κ_M_ = 0.755 before and κ_M_ = 0.979 after feedback, an increase from substantial to almost perfect agreement.

**Table 2:** Third coding level.

Level 3 – Mental micro activities		Description	Examples
1. Proactive / focused / self-assertive (A) – PF-A		Focused attention; investigative observation; proactive, extroverted, and initiative attitude in the interaction; person A is aware of herself and her intentions regarding B and thus may express herself self-assertively.	“… First, a consideration of face, shape of nose, mouth, eyebrows, eyes, the face as a whole thinking, observing, being with me and looking at her” (P 23)
2. Proactive / focused / self-assertive (B) – PF-B		Analogous to Cat. 1 with roles reversed; from the perspective of person A, a proactively focused attitude emanating from person B is perceived; one feels looked at, fixed, or challenged; the intention directed at one can be experienced as the self-assertion of person B; uncomfort feelings or escape impulses can arise	“… She was switching back and forth between my two eyes the whole time” (P 10) “The long eye contact feels like a ‘nakedness’, as if you are giving the other person a glimpse of your inner self” (P 19)
	2.1 PF-B protection	Only mask condition: Person A perceives her mask as a protection from PF-B activities or as a means of hiding her facial expressions from the other person’s gaze; this can be accompanied by positive, especially relieved feelings.	“With mask I felt more comfortable and confident in the encounter, eye contact was immediately easier” (P 13)
3. Receptive / opening / devotional (A) – RO-A		Person A opens up to, identifies and accepts what is coming from person B as described in Cat. 2; an inner space is given for what emanates from person B and is perceived receptively; person A surrenders to B’s presence without asserting their own impulses at this moment.	“It was as if I opened myself for a moment and made myself empty, so that the impression of the other person in me could get space” (P 13)
	3.1 RO-A inhibition	Only mask condition: Person A perceives the mask of person B as obstacle for her own RO-A activity and experiences a reduced empathy. Typical expressions include the “depth” or “soul” of the other person, which cannot be adequately grasped.	“The experience of being able to look through the eyes into the depths of the partner was lost” (P 22)
4. Receptive / opening / devotional (B) – RO-B		Analogous to Cat. 3 with roles reversed; person A experiences person B opening to and receptively receiving what emanates from herself (A); person A feels perceived, accepted, or secure; person B is perceived as being with person A and suspending her own intentions.	“The feeling of being noticed” (P 29) “She also noticed that in my eyes and had also gotten ‘tunnel vision’” (P 32)
5. Connection / resonance			
	5.1 Positive	Felt connection or resonance between the participants; binding exchange, which is less static, but rather exhibits a subtle and bidirectional dynamic; more general or symmetric than categories 1 to 4; this can be accompanied by feelings of agreement, trust, closeness, or intimacy.	“In the end, I had the impression that we were on the same wavelength” (P 20)
	5.2 Negative	The opposite of category 5.1; typical expressions are negations of connection, diminished contact, interaction difficulties, distance, isolation, and feelings of coldness or antipathy.	“…however, I felt that moment as if there was a distance between us” (P 5)

Coding categories with subcategories, descriptions, and exemplary excerpts from the data.

In addition, we performed an (half-) automated qualitative analysis to investigate the (in) dependence of Level 3 categories on body reference, as also addressed by our first hypothesis. To identify body reference in the data, a list of corresponding terms was compiled, and all coded segments were searched for the occurrence of at least one of these terms. In fact, to cover all body references in Level 3 codings, the following terms sufficed: body, exterior, eye, face, mask, to view/see (all forms, only physical context). Since the term “gaze” is ambiguous regarding body-related and mental contexts, this was treated as an extra category. The remaining codings contained neither a body reference nor the term “gaze.” Below, exemplary excerpts from the data are presented (see section “Results”) and discussed in qualitative or phenomenological regard (see section “Discussion”).

#### Code relations analysis

Code relations include not only the already mentioned overlapping of categories in coded data, but also topological aspects of code distribution in the protocols. Since at Level 1 the data were almost completely coded with two categories and without overlap, we investigated the distribution of dyadic and monadic passages in the data sets per participant and condition to explore whether specific patterns could be detected. With the “document portrait” tool in our qualitative data analysis software (MAXQDA), we found periodic changes between dyadic and monadic sections in most data sets, which were then quantitatively analyzed regarding the frequency of changes per protocol and compared between the experimental conditions.

Regarding overlapping coding, initially intralevel relations are analyzed more precisely before addressing interlevel overlaps. Aggregation of frequencies of overlapping codes in a cross table with selected categories (“Code Relations Browser” in MAXQDA) allows one to find and evaluate the most pronounced code relations. Following the approach of Krikser and Jahnke (2021), the analysis was limited to an interval from the maximum of category overlap to its half. At Level 2, the maximum of seven overlaps occurred between “Attention Regulation” and “Intention,” five between “Thought” and “Affective Feelings,” and four between “Eye Gaze” and “Affective Feelings” (see Table 1). Regarding the former, this indicates a subtle connection between the categories as in these segments aspects of attention regulation were not only reported but also intentionally controlled at a metacognitive level. The latter two relations refer to different aspects of feeling, firstly as reflecting emotions more intellectually, and secondly as immediately experienced feelings in dyadic gazing. At Level 3, two segments were identified in which the proactive-focused gesture was reported as equally referring to both partners, and in one segment this occurred for the receptive-opening gesture.

**Figure 1:**
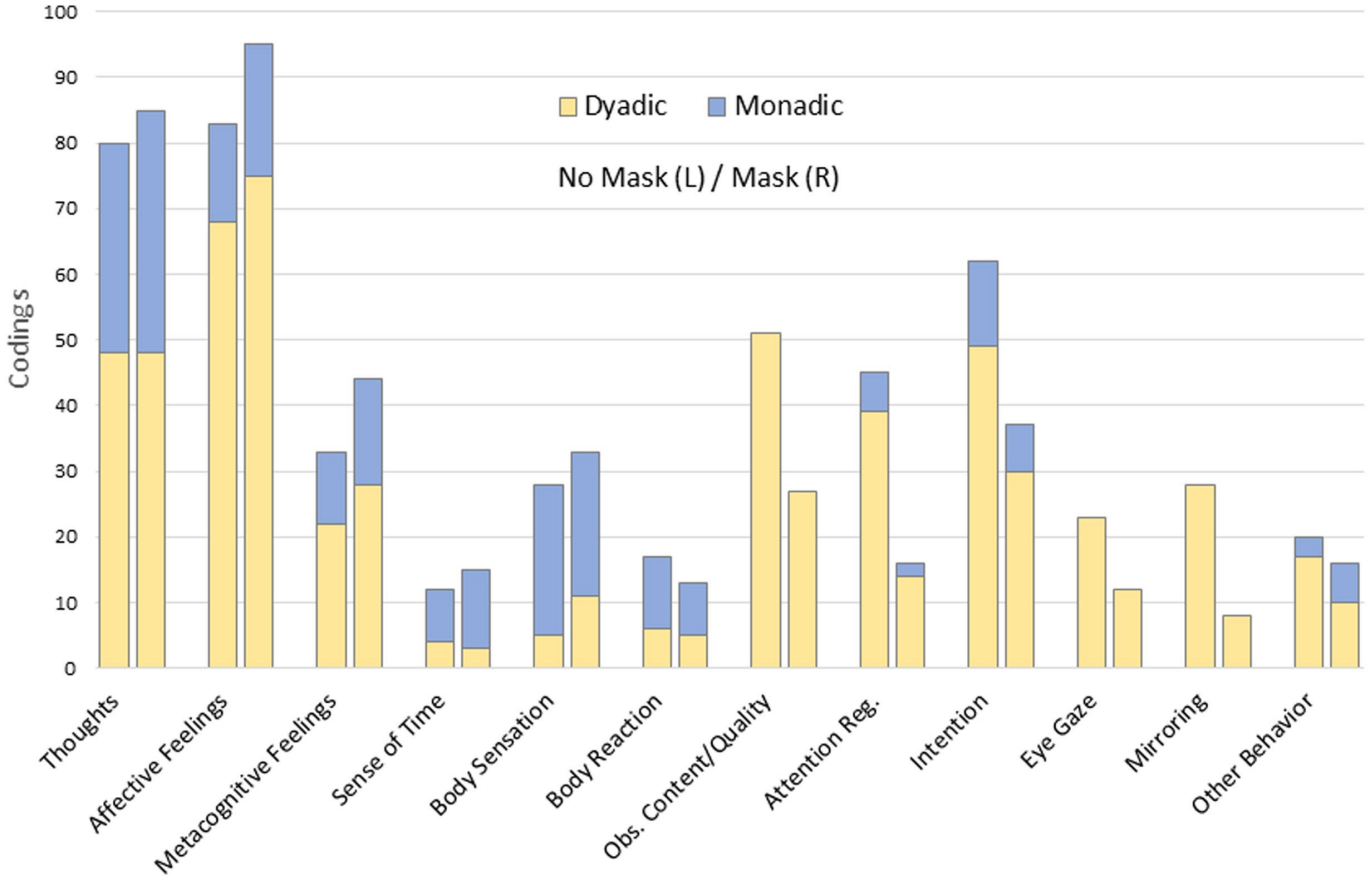
Code relations between levels 1 and 2.

**Figure 2:**
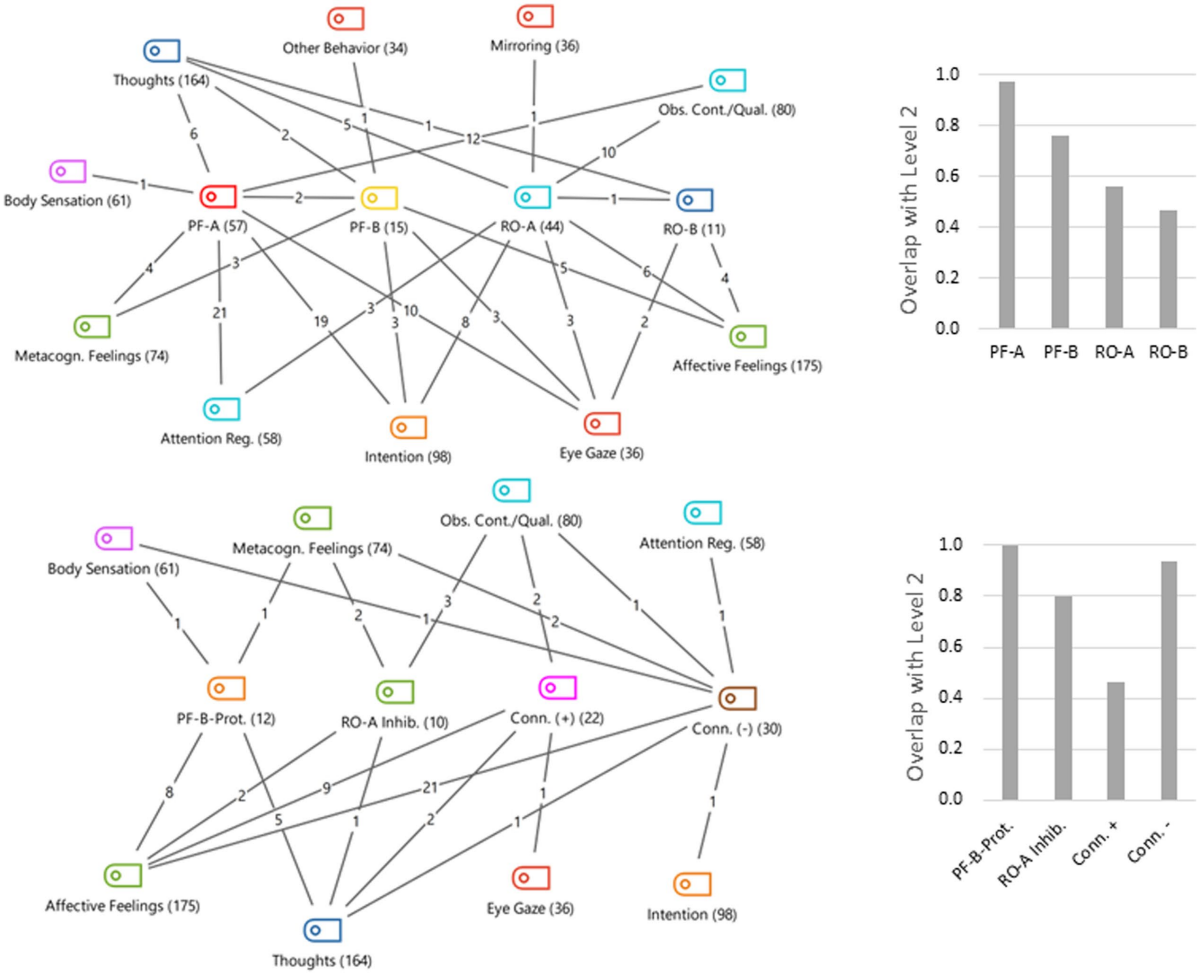
Code relations between levels 2 and 3. Level 3 categories are placed in the middle of the code maps, surrounded by level 2 categories that overlap with them. The total frequencies are in brackets, the overlaps are on the lines. From interlevel relations, only those for level 3 are shown. The diagrams show the portion of overlap between level 2 and level 3 categories.

Coming to interlevel relations, it is not surprising to find almost complete overlaps between Level 1 and each of the other two levels, which we have broken down for the individual categories. While intersections between Levels 1 and 2 show mixtures of dyadic and monadic forms of experience in most categories (Figure 1), codings at Level 3 coincide entirely with dyadic experience, apart from just two segments in which the receding or lack of connection (still dyadic) transforms into a purely self-referential experience (monadic). For Level 2 in most, but not all, categories dyadic experience predominates, and there are individual (almost) purely dyadic categories, which will be discussed in more detail below. Considering Levels 2 und 3 together, it turned out that 95% of the data was covered by them, which means that Level 3 covers 6% of the 11% that were missing at Level 2, and that 17% of Level 3 encodings are reinterpretations of Level 2 encodings (interlevel overlap). The 5% of the data that remain uncoded consist of unclear, ambiguous, contradictory statements (in the context of Levels 2 and 3), or statements not related to the task or including blanks and paragraph marks that were not coded. An overview of the qualitative and quantitative aspects of overlapping between Levels 2 and 3 is given in Figure 2.

#### Statistical analysis

Some quantitative aspects of the protocol data were examined independently of the qualitative analysis, such as protocol length and word frequencies (e.g., first-person pronouns). In the comparison of such measures with other work or between experimental conditions, first indications were discussed in the light of the findings of the qualitative data analysis. Another instance of this is the automated search for body-related words in Level 3 data, which we conducted after qualitative coding but methodologically independently of it, as explained below (see section “Results”). Most statistical investigations, however, were based on the two qualitative analysis steps described above. Here, two variants must be distinguished, the first of which operates with frequencies of coded segments per data set transforming categories into metrical variables. In contrast, for the second variant quantities of coded segments are binarized depending on whether a category occurs in a protocol or not, leading to nominal variables (binary occurrence of categories in data sets) or metrical variables (number of coded categories per data set). The first variant was only applied to Level 1, where data were coded with two dichotomous categories whose portions can be determined for each data set and compared for differences between the conditions by t-tests. For the second variant, nominal variables were investigated by chi-square tests backed up by an exact test for frequencies below five (Boschloo, 1970), and metrical variables were again examined by t-tests. To control for the family-wise error rate in multiple testing, we used the sequential Bonferroni method developed by Hochberg (1988), which is still relatively conservative in terms of maintaining the null hypothesis (Cramer et al., 2016). Code frequencies were also compared with those of a previous study (Wagemann and Weger, 2021) to check whether and to what extent the mental activity structure suggested by Level 3 categories could be replicated.

## Results

The presentation of results begins with purely quantitative aspects of the protocol data and then follows the hierarchical structure of Levels 1 to 3 with qualitative and quantitative results. While the former results serve to identify initial clues in terms of the hypotheses, those for Level 3 aim directly at the detectability of specific mental micro-activities, their dependence on overt bodily behavior, and their susceptibility on the experimental conditions, as will be shown below.

### Protocol length and first-person pronouns

Initially, the quantity of written words and portions of first-person pronouns (I, my, me) per data set were compared for the experimental conditions. There was a significant decrease in protocol length for the interaction task without mask (*M* = 176.7, *SD* = 73.7) compared to interaction with mask (*M* = 136.6, *SD* = 65.9), *t* (33) = 4.5, *p* < 0.001, *d* = 0.75. Comparison of first-person pronouns yielded no significant difference between the conditions, *t* (33) = 0.51, *p* = 0.612, despite without mask (*M* = 9.2%, *SD* = 0.038) the percentage was slightly higher than with mask (*M* = 8.9%, *SD* = 0.044).

### Dyadic and monadic forms of experience

**Figure 3:**
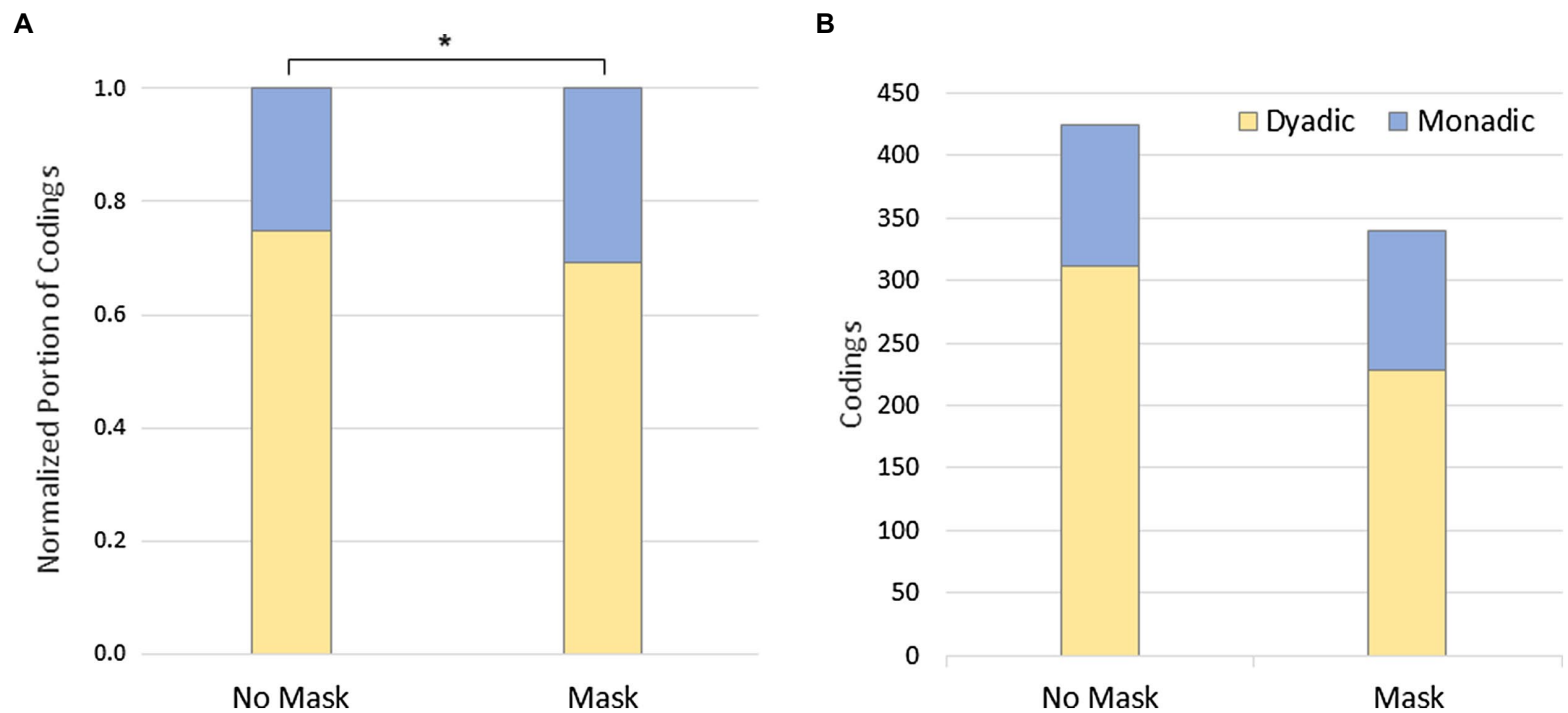
Level 1: Dyadic/monadic experience. **(A)** Relative rating, *p = 0.048; **(B)** absolute rating.

Building on qualitative analysis, at Level 1 the portions of dyadic/monadic codings normalized per data set were compared for the experimental conditions (Figure 3). A one-tailed t-test seems to strengthen our second hypothesis that dyadic experience is stronger without mask (*M_Dyadic_* = 74.8%, *M_Monadic_* = 25.2%, *SD* = 0.193) than with mask (*M_Dyadic_* = 69.2%, *M_Monadic_* = 30.8%, *SD* = 0.182), *t* (33) = 2.05, *p* = 0.048, *d* = 0.30. In absolute terms, only the number of dyadic codings per data set decreased from *M_No Mask_* = 9.1 to *M_Mask_* = 6.7, while the number of monadic codings remained almost constant across the conditions, *M_No Mask_* = 3.32, *M_Mask_* = 3.24 (Figure 3B). However, to disentangle this from the mentioned reduction in protocol length, we restrict the test to a consideration of the portions. A second quantitative result for Level 1 concerns the frequency of changes between dyadic and monadic passages in the data sets yielding *M_No Mask_* = 3.32 and *M_Mask_* = 3.21, the difference of which was not significant, *p* = 0.409. The fact that these values are quite close to those for monadic coding may be coincidental, since monadic/dyadic passages partly contain more than one appropriately coded segment.

### Multiple aspects of first-person experience

**Figure 4:**
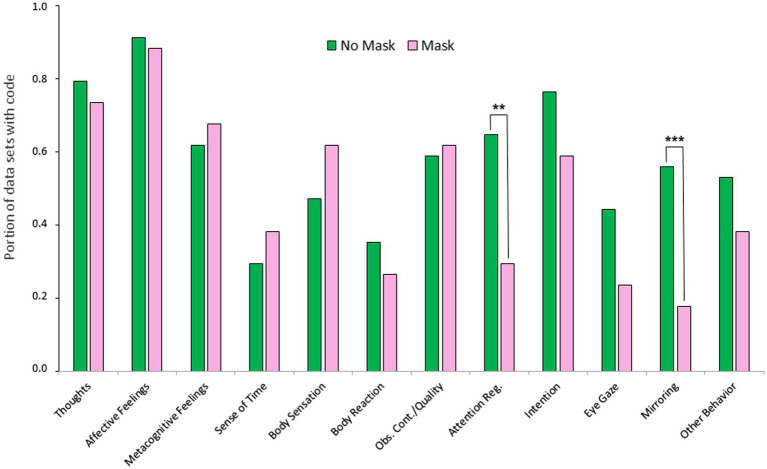
Level 2: Multiple aspects of first-person experience. **p =0.007, ***p < 0.001, all others not significant, *p* > 0.072.

For statistical analyses at Level 2, as said, coding quantities were binarized per data set and investigated regarding the number of coded categories per data set and, conversely, the portions of data sets containing certain codes. Firstly, the number of coded categories per data set decreased significantly from interaction without mask (*M* = 7.0, *SD* = 2.2) to interaction with mask (*M* = 5.9, *SD* = 1.8), *t* (33) = 3.68, *p* < 0.001, *d* = 0.64, indicating that self-reports under mask condition were not only shorter but also less differentiated in terms of Level 2 categories. Secondly, with a chi-square test, significant differences in code frequencies across the conditions were found for Attention Regulation, *χ^2^* (1, *N* = 68) = 7.2, *p* = 0.007, *w* = 0.32, and Mirroring, *χ^2^* (1, *N* = 68) = 12.6, *p* < 0.001, *w* = 0.43, while all other differences were not significant (see Figure 4). This reduction in certain categories can be understood as a concretization of less differentiated self-reports under the mask condition.

### Mental micro-activities

For Level 3, we first provide some examples of coded data for phenomenological illustration (in addition to Table 2), as this is conceptually central to the study. At the same time, the above-mentioned double codings that occurred here for PF-A/B and RO-A/B shall be elucidated. While without mask, the segment of “Both participants kept trying to focus their gaze noticeably” (P 06) was coded under PF-A/B, with mask it was rather a metaphorical expression: “It’s like a picture again … as if we are looking over the garden fence, secretly watching each other” (P 08). For RO-A/B, the segment of “The person in front of me and myself was ‘open’” (P 31, without mask) immediately illustrates the contrast to the complementary gesture.

**Figure 5:**
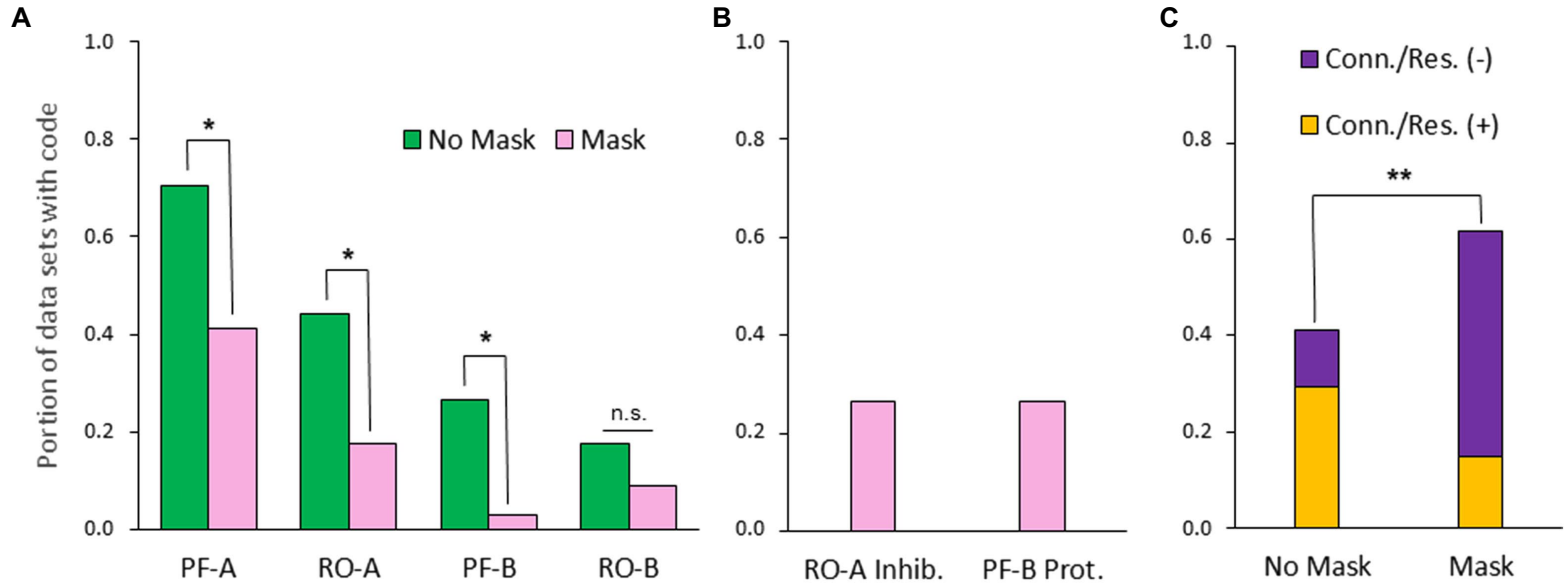
Level 3: Micro-activities. **(A)** The four micro-activities (PF: Proactive-focused, RO: Receptive-opening), reordered by size, *p < 0.019, n.s., not significant, *p* > 0.39. **(B)** Negative aspects related to micro-activities (inhibition, protection, only mask cond.); **(C)** Connection/Resonance (+/−), **p = 0.0082.

**Figure 6:**
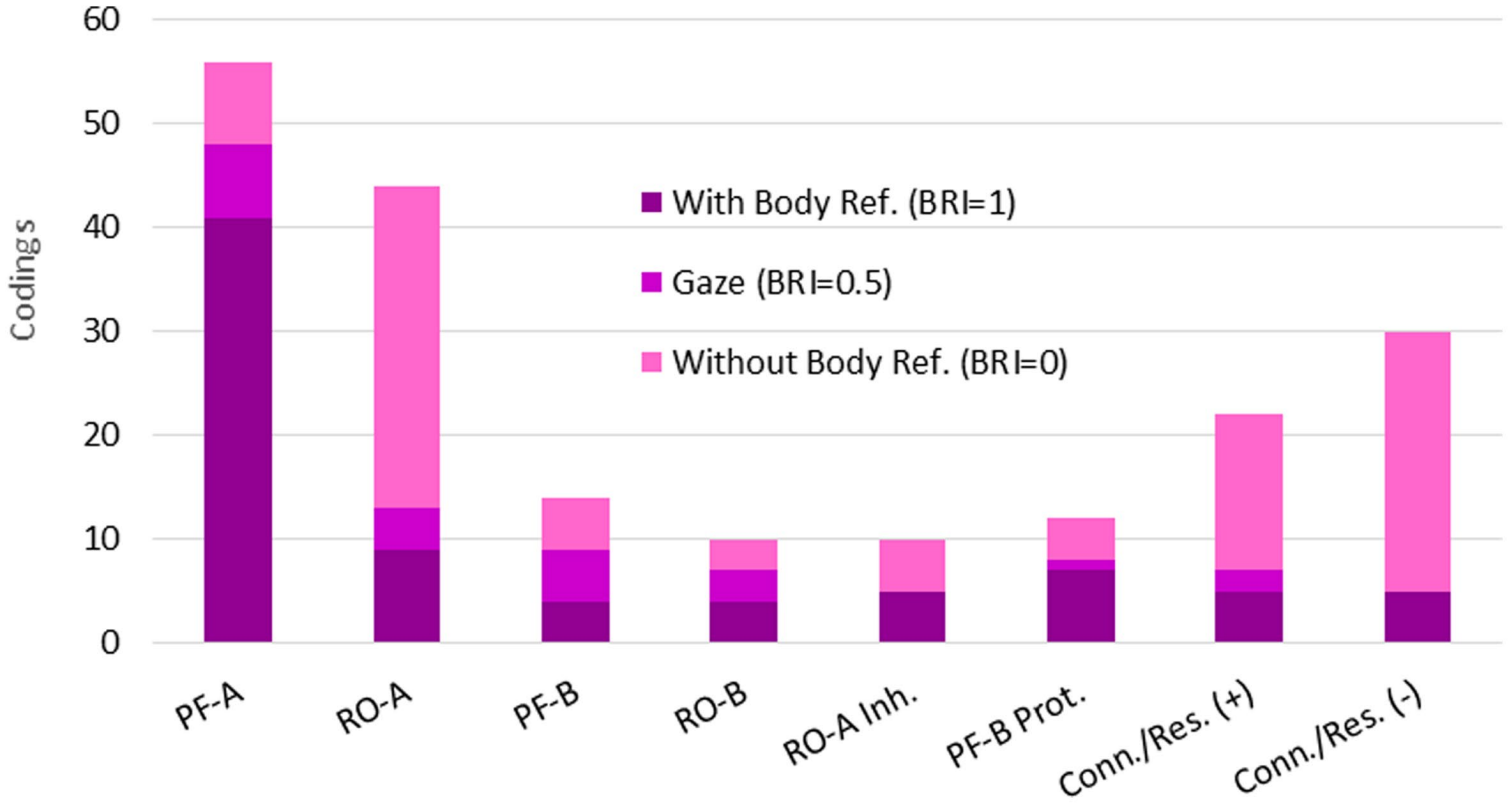
Level 3: Body reference in mental micro-activities.

Turning to the quantitative results at Level 3, the number of coded categories per data set did not change significantly (*M_No Mask_* = 2.1, *M_Mask_* = 1.9, *p* = 0.36), which could also be because two additional (negative) aspects occurred under mask condition that were not relevant for the other condition (RO-A Inhibition, PF-B Protection, Figure 5B). Remarkably, in view of both hypotheses, most variables decreased significantly from interaction without mask to interaction with mask, even after correction for multiple testing (see Table 3), which will be discussed below as a crucial point. To test the dependence of micro-activities on overt body behavior, automated text analysis at Level 3 yielded 80 segments (out of 47 data sets) with unambiguous body reference, 23 (out of 19 data sets) containing “gaze,” and 98 (out of 38 data sets) without explicit body reference, the distribution of which across the categories is shown in Figure 6. Examples of the coded data subdivided into these three aspects are shown in Table 4. To characterize the individual distributions, we calculated for each category a *body reference index* (BRI) by weighting segments with body reference by 1, those with gaze reference by 0.5, and all without explicit body or gaze reference by 0 and averaging them accordingly (Figure 6; Table 5). The difference between PF-A and RO-A (independent of conditions) proved highly significant with large effect size in a Chi-square test between BRI = 1 and BRI = 0, *χ^2^* (1, *N* = 89) = 33.5, *p* < 0.001, *w* = 0.61, indicating that PF-A is strongly associated with body reference, while RO-A is not. While the mean values suggest a slight predominance of lacking body reference in both conditions, it must be considered that they are derived from the numbers of the coded segments. To balance this with a participants-based measure, BRI was also calculated for each data set, averaged over Level 3 categories. In this way, we obtained a metrical variable that could also be examined across the experimental conditions. For this purpose, however, only data sets from 28 participants with segments coded at Level 3 *in both conditions* could be used for a dependent-samples t-test. The mean values resulting from this procedure show, in contrast to the above consideration, a slight predominance of body reference, *M_No Mask_* = 0.60 and *SD_No Mask_* = 0.32, *M_Mask_* = 0.55 and *SD_Mask_* = 0.34, whose slight decrease over the conditions was not significant, *t* (27) = 0.85, *p* = 0.401. The relatively high standard deviations indicate a high interindividual variability of BRI.

**Table 3:** Level 3: Differences across conditions for portions of data sets with code (binarized).

	PF-A	RO-A	PF-B	RO-B	RO-A Inh.	PF-B Prot.	Conn./Res. (+)	Conn./Res. (−)
No mask	0.706	0.441	0.265	0.176	0	0	0.294	0.147
Mask	0.412	0.176	0.029	0.088	0.265	0.265	0.118	0.471
*p*	0.015	0.018	0.007	0.396	0.001	0.001	0.008	
Test statistic	*χ^2^*(1, *N* = 68) = 6.0	*χ^2^*(1, *N* = 68) = 5.6	Exact test (Boschloo)	Exact test (Boschloo)	Exact test (Boschloo)	Exact test (Boschloo)	Exact test (Boschloo)	
*w*	0.3	0.29	0.33	0.13	0.39	0.39	0.48	
Adj. *p*	0.036	0.037	0.033	0.396	0.005	0.005	0.033	

An exact text according to Boschloo (1970) was used when absolute frequencies occurred below five. The last row shows adjusted *p* values according to Hochberg (1988). Significant results are highlighted.

**Table 4:** Level 3: Varying dependence on body reference (exemplary codings).

	With explicit body ref.	Gaze (BRI = 0.5)	Without explicit body ref. (BRI = 0)
	(BRI = 1)		
PF-A	I start to look more closely at the person’s face covering. (02/with)	… so that I had the eyes in focus as if in tunnel vision (05/without)	I wanted to be closer to you again and I formulated my feelings and sensations that came up at that moment to you. (28/with)
RO-A	… look into her eyes, be open to her (be with her) (23/without)	My partner returned my gaze, but she felt very uncomfortable under observation. (13/without)	I open up to the person and feel strongly involved and responsible. (17/without)
PF-B	She switched back and forth between my two eyes the whole time. (10/without)	I also wanted to avert my gaze for a second, because it had become very exhausting to hold the gaze toward the end. (26/without)	I feel naked, exposed (08/without)
RO-B	… I had the feeling she opens her eyes to me and with that also the inside, so that I can look into her soul and she has nothing to hide from me. (10/without)	She also noticed that in my eyes, she also got a “tunnel vision” and she noticed that my thoughts are completely with me… (32/with)	… the feeling of being perceived (29/without)
RO-A Inhib.	The experience of being able to look through the eyes into the depths of the partner was lost. (22/with)	–	I do not experience you. (14/with)
PF-B Prot.	The mask was like a “protective shield” (27/with)	With mask I felt more comfortable and confident in the encounter, eye contact was immediately easier. (13/with)	I did not feel watched and much more relaxed. (10/with)
Conn./Res (+)	We both realized that this task is very personal right now and the eye contact is very profound. (32/without)	The look represents a connection, almost a dependence. (17/without)	I felt a connection between us. (18/with)
Conn./Res. (−)	The emotional distance was greater than in the first trial, despite the strong eye contact. (07/with)	–	Didn’t feel any connection between us. (20/with)

Information about participant and condition is indicated in brackets.

**Table 5:** Level 3: Body reference index across categories and conditions.

	PF-A	RO-A	PF-B	RO-B	RO-A Inh.	PF-B Prot.	Conn./Res. (+)	Conn./Res. (−)	*Mean*
No mask	0.800	0.264	0.462	0.500	–	–	0.235	0.286	0.422
Mask	0.786	0.188	0.500	0.667	0.500	0.625	0.400	0.130	0.451

In view of our hypotheses, we further narrowed the analysis on Level 3 to the four micro-activities (PF-A/B, RO-A/B), accumulated them as a subcategory, and investigated its overlaps with the three levels of BRI as well as with the Level 2-category of *intention*. Remarkably, every test yielded significant differences across the conditions (even after correction for multiple testing) with medium effect sizes, with the largest results for overlap with intention and with BRI = 0 (see Table 6).

**Table 6:** Overlap of accumulated micro-activities (Level 3) with body reference index (BRI) and intention (Level 2) across conditions.

	Four micro-activities (PF-A/B, RO-A/B)			
	BRI = 0	BRI = 0.5	BRI = 1	Intention (Level 2)
No mask	0.471	0.353	0.676	0.441
Mask	0.176	0.147	0.412	0.147
*p*	0.01	0.05	0.028	0.008
*χ^2^*(1, *N* = 68)	6.7	3.8	4.8	7.1
*w*	0.31	0.24	0.27	0.32
Adj. *p*	0.029	0.05	0.05	–

The last row shows adjusted *p* values according to Hochberg (1988). Significant results are highlighted.

**Figure 7:**
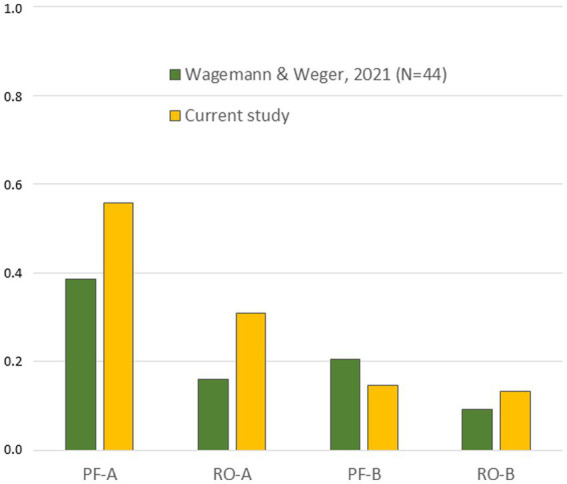
Comparison of mental micro-activities between studies. Frequencies were averaged over experimental conditions for both studies. All differences were not significant, *p* > 0.12.

Last but not least, against the background of replicability (Hypothesis 1), frequencies of the four mental micro-activities were compared between the current study and prior work. In Wagemann and Weger’s (2021) study, participants were instructed to move around for 5 min in a group of 22, nonverbally form dyadic pairs, and interact nonverbally for a short period of time before separating again and moving on to further encounters. In a between-subjects design, one group was forbidden to engage in physical contact such as hugging or hand shaking while the other group did the task without these restrictions. Frequencies averaged across conditions from both studies are shown in Figure 7. Except for PF-B, similar patterns sloping from PF-A to OR-B emerge, all differences were not significant, *p* > 0.130.

## Discussion

### Summary and evaluation of results

First, we summarize the results and draw initial conclusions for the hypotheses before discussing this in broader psychological and philosophical contexts. The purely quantitative results already contain some clues whose traces can be followed through the various levels of further analysis. Regarding the variation of protocol length across the conditions, we must of course be cautious in that it could be a fatigue effect due to the sequence and that perhaps, in the second condition, participants focused on noting differences from the first condition rather than re-describing repetitive experiences. Conversely, however, the order of conditions could have led them to write more in the mask condition through practice and sensitization in the first phase (Greenwald, 1976; Charness et al., 2012). Considering this ambivalence, one should neither overestimate nor ignore the significantly longer protocols without mask, also because we found such differences in a between-subjects design on another first-person topic which could be well explained theoretically (Wagemann, 2022b). So, apart from carry-over effects, a possible explanation would be that there was less to observe and describe under the mask condition than without mask, which seems to be supported to a slight extent by the reported variation of first-person pronouns. In any case, the fact of the overall high occurrence of first-person pronouns, as confirmed by other work (Chung and Pennebaker, 2007; Seih et al., 2011), suggests that the participants followed the instructions correctly and reported from the first-person perspective. Apart from this, as a first qualitatively grounded aspect, the finding of significantly more differentiated descriptions at Level 2 without mask supports the explanation of an impoverished experiential field under mask condition.

As a further concretization of this tendency, the significant shift of the portions of dyadic and monadic experience can be mentioned, which is based on a reduction of dyadic experience while monadic experience remained almost unchanged. Together with the finding of a nearly constant alternation between the two forms of experience in the protocols, this suggests that the dynamic has shifted toward the monadic pole. If we pursue this issue further at the second coding level, we find that the two significantly decreasing categories are almost exclusively (Attention Regulation) or exclusively (Mirroring) dyadic in origin. In fact, these categories represent two core aspects of dyadic experience, as Attention Regulation refers to Person A’s mental capacity to control various forms of attention in the encounter, while Mirroring reflects the embodied resonance of this capacity in the dyadic interaction involving both persons. Similarly, as the rather general effect on Level 1 could be concretized at Level 2, these two sides of dyadic experience – more and less embodied – can be fleshed out even more clearly on Level 3. Investigation on Level 3, differentiated as on level 2, but focused on mental micro-activities, leads to seven significantly changing categories out of a total of eight (if the categories occurring only under mask condition are counted). Thus, it is probably not too much to claim that the core effect emerges here, which is already implied at the other levels of analysis. However, while code relations analysis between Levels 2 and 3 revealed partial overlap of Attention Regulation with self-centered Level 3 categories (PF-A, RO-A), Mirroring almost did not coincide at all with these forms of mental activity and experience (Figure 2). This could be interpreted as distinguishing embodied dimensions of dyadic interaction and experience (e.g., Mirroring) from more subtle forms of inner behavior and expression that are, at least to some degree, independent of the former. According to the results for the body reference index (BRI) on Level 3, the idea of graded forms of embodied experience is concretized for mental micro-activities which, in this regard, does not seem to change their composition across the experimental conditions. Overall, in retrospect, we believe that the various interlocking dimensions of our methodological approach are justified by the rich and nuanced results.

In view of the hypotheses stated above, we can draw the following conclusions. First, the occurrence of mental activity differentiated in the mentioned two or four forms, respectively, is accounted for by the reliable coding at Level 3, which thus replicates the basic finding of Wagemann and Weger (2021). That these micro-activities are likely to play an important explanatory role in social interaction is further suggested by their significant susceptibility across the experimental conditions (without mask/with mask). Regarding the relationship of PF-A/B and RO-A/B to overt and covert behavior, the presumed ambivalence could be supported by an approximately equal distribution of body-related and inner-mental expressions, which seems to be unchanged across conditions. While in the former case (BRI = 1) participants’ experience can be characterized as “strongly embodied,” in the latter (BRI = 0) we suggest speaking of “weakly embodied” experience, indicating a certain independence of mental activities from overt or bodily behavior, which should not imply here any final ontological view on the body–mind problem (see Menary, 2015). The second hypothesis about decreasing frequencies of the four micro-activities was confirmed and additionally strengthened by the unexpected occurrence of deficit-related statements (PF-A Protection, RO-A Inhibition) and by the significant decrease in holistic relationship quality (Connection/Resonance +/−).

### Theoretical implications

While the empirical hypotheses are substantiated by the results, the theoretical and philosophical implications remain to be developed. Here, we go beyond hypothesis testing and introduce the integrative concept of an I-Thou sense, as indicated in the title of this study, whose applicability to the empirical results only became apparent during data analysis. To realize this key aspect justified in the methodological scope of our mixed-method approach, as indicated in the introduction, direct social perception (DSP) shall be focused upon here based on some preparatory considerations. Firstly, the notion of shared attention will be extended to clarify the structural role of two “quasi-” reference objects in nonverbal social interaction, one bodily and one mental. Secondly, as DSP accounts are challenged to explain in what sense social perception might be direct, the definitional criteria of a *sense* will be examined in order to determine to what extent they could justify a quasi-sensory tool (e.g., I-Thou sense) that goes beyond the commonly known modalities. Then, on this basis, some similarities and differences between our account and DSP are discussed and finally connected with the aspect of face masks.

#### Reference “objects” in shared dyadic attention

For the first step, we start by distinguishing *shared attention* from *joint attention*, both of which are often used synonymously. Joint attention refers to situations where one individual (A) follows the gaze of another (B) directed toward a reference object, with B unaware of A’s attentional state. Shared attention, in contrast, also includes mutual gazing between the individuals, transforming the situation into a symmetrical one (Emery, 2000; Stephenson et al., 2021). Hence, our experimental setting could be understood in some sense as promoting an unambiguous case of shared attention, although the common reference object is questionable due to the lack of additional task content. Here, we must distinguish between the two basic forms of mental micro-activity. When person A exerts proactive-focused activity directed toward the other, the shared reference object seems to be located in person B’s physical appearance, as is evident from the high body reference index of PF-A (Table 5) and from PF-B Protection under mask condition. In contrast, when A exerts receptive-opening activity, it is not the mere physical appearance of B, but B’s expression through it in the form of mental activity that stands out, so to speak, as a shared reference object – or “subject-object.” While this is empirically supported by the significant decrease in BRI from PF-A to RO-A, the data coded under RO-A Inhibition (mask condition) show that this activity is not completely independent of body reference. Thus, transferring shared attention from its generic triadic context (two subjects, one object) to a “purely” dyadic setting (two subjects without additional object), it is necessary to distinguish two different types of “quasi-” reference objects, a physical-bodily one and a mental one, which are experienced differently (strongly vs. weakly embodied). Moreover, it must be considered that both types of reference objects are not independent of interactants but are related to them and also expose complementary connections to proactive-focused and receptive-opening activity. In particular, the aspect of activity-related reference *objects*, which, from their origination, are also *subjects* (“subject-object”), suggests the need to broaden this modified notion of shared attention into what Michael Tomasello calls *shared intentionality* (Tomasello et al., 2005). Since shared intentionality is understood as goal-directed collaborative behavior, the overarching goal of participants can be seen in the joint performance of the task, while process-related goals can be seen in the coordination of specific forms of mental activity. That intentions and goal hierarchies are indeed relevant here in the context of mental activities is concretized empirically by the significant decrease in overlapping portions between conditions (Table 6). Philosophically, this can be linked to the mental action debate, in which conscious intentions behind mental activities are discussed as criterion for mental action (O’Shaughnessy, 2000; Proust, 2001; Peacocke, 2007), but we cannot pursue this here for reasons of space.

#### Aspects of a sense of I and Thou: What kind of information?

Next, as DSP accounts focus on non-inferential, “cognitively impenetrable” processes in early stages of sensory processing (Krueger and Overgaard, 2012; Michael and de Bruin, 2015, p. 250), one might ponder whether this attentional or intentional structure is a sense in itself. Of course, regarding the huge amount of empirical and theoretical research on the constitution of a sense modality, we cannot claim that our findings establish the existence of a hitherto unknown sense. Nevertheless, we do think that the evaluation of the self-reports suggests that further research in this direction could be fruitful. Why is this so?

In order to be able to speak of a sense, at least some definitional criteria, which are mostly accepted in this field of research, must be fulfilled: (1) A sense provides access to information about the external environment or our own condition, (2) each sense has its own organ with corresponding receptors and responds to characteristic stimuli, and (3) can be described by specific forms of processing that are usually thought to unfold along neural pathways leading to specific brain areas (American Psychological Association, 2022). Although this is a simplified version of more differentiated definitions of a sense modality (e.g., Matthen, 2015), it is useful as a first step. To begin with the first point, information referring to the dyadic interaction partner is highly differentiated at a physical level, as described in Level 2 categories (4.1 Observation content/quality, 6. Behavior and interaction), but structurally integrated in terms of the two characteristic mental activities on Level 3. Here, only the PF-B and RO-B activity is what person A captures as coming from person B. Whereas Level 2 information might be interpreted in the Theory of Mind as a basis for inferences about multiple mental states of the other or as an occasion for simulating their differentiated emotions or external behaviors, Level 3 information provides just the two mentioned forms of mental activity that are likewise exercised by oneself. In this respect, beyond one’s own activity dispositions, there is no content at all that could presumably be inferred or simulated, and instead of deploying *this content* for ascriptions of propositional attitudes to the other, nonverbal interaction is about shaping the dynamics of attentional movement corresponding to it. So, why should not there be a sense modality that refers to the *exertion and experience* of one’s own and others’ mental activity, analog to active and passive kinesthesis (as, e.g., in interpersonal touch or dancing, Kronsted and Gallagher, 2021) conveying sensations about intentionally moving or being passively moved, except that here it is not about physical but about mental (self-) movement? As our studies on visual and auditory perceptual reversal have shown, the exercise of productive and receptive forms of mental activity plays a crucial role for these conventional modalities too (Wagemann et al., 2018; Wagemann, 2022). Thus, for two subjects interacting nonverbally and without prescribed social or communicative content, this suggests the reconstruction of a sensory modality that precisely relates to the dynamics of proactive and receptive mental movement otherwise employed in object-related perceptual or other cognitive processes.

#### Aspects of a sense of I and Thou: Which receptors?

Concerning the second aspect of sense, our above considerations have shown that, while physical sense organs are certainly needed to provide stimuli referring to external expressive behavior (PF-A), their relevance is attenuated when it comes to identifying the expressor or mental agent standing behind and permeating their bodily conveyed utterances (RO-A). Put differently, the bodily sense modalities (e.g., vision, audition) serve as a necessary but not sufficient condition for “perceiving the other self” (Wagemann and Weger, 2021), being periodically suspended and transcended to some degree (as indicated in low values of BRI) to give access to an experience of the other’s mental movements. This is not to be misunderstood as the other’s mental activity being perceived (e.g., “seen”) *in* the bodily expression; rather, the latter becomes transparent to the activity that may be triggering it or proceeding covertly without external expression. This antagonistic relationship between the phenomenal prevalence of bodily expression and mental expressor (see Table 4) suggests that, in this special case, sensory stimuli and receptors could be of the same kind – mental activity – and differ only in their complementary form and the individual in whom they are enacted at a time. Then, stimuli would be the individually exerted forms of mental activity by one person while portions of receptive-opening activity performed by the other would act as receptor. Although this conception of stimulus and receptor may seem quite unusual at first glance, it simply reflects at a mental level the natural equivalence of physical or chemical stimuli and correspondingly adapted physiological receptors in other modalities. While an acceptance of this idea ultimately depends on the status of reality one is willing to grant to mental versus material phenomena, it finds support in a pragmatist-interactionist approach to aesthetic perception, as will be briefly outlined. Instead of locating aesthetic qualities in certain perceptual features of objects (externalist account) or in detachment from perceptual and pragmatic intentions and the achievement of pure intuition (transcendental account), they can be found in *indications of potential interactions* an observer may have with an object (Bickhard, 1993; Xenakis and Arnellos, 2014). In this perspective, aesthetic experience is stimulated by affordances of the environment, i.e., perception-action relationships which do not require prior knowledge to become effective and can (but need not be) perceived by the individual (Gibson, 1979). Importantly, interactive affordances and their responsive realization are not limited to art-centered contexts, but are inherent to everyday life (Dewey, 1980; Shusterman, 2010; Xenakis and Arnellos, 2014). Applied to our situation, it is not the externally (sometimes only subliminally) perceptible expressions that form the “social-aesthetic” experience of the interaction, but the specific affordance character of mental activities and according responses. The other responds to these affordances with reciprocal or complementary forms of their own mental activity, so that both are connected in a “mental behavior loop” constituting a dynamic relation of stimulus and receptor, which can be also characterized by the resonance metaphor (Shepard, 1983; Gedenryd, 1993).

#### Aspects of a sense of I and Thou: Which processes?

Thirdly, regarding the processual aspect of sense, our empirical findings and theoretical considerations suggest that, in contrast to the accompanying neural processes (e.g., the mirror neuron system, see above), the core dynamics can be reconstructed without leaving the first-person perspective. Although there is almost no immediate evidence in the data for an unambiguous sequential order of the forms of activity, such an order seems logical due to their complementarity and is precisely described by Steiner (1964, 1966). For example, let us start with person A scrutinizing person B’s physical appearance with interest (PF-A). In this case, B is likely to feel physically looked at, which implies a certain level of receptivity and may in turn be registered as RO-B from A’s perspective. After a short time, however, this receptivity changes into proactive-focused activity on B’s part, as is supported by studies on endurance of the gaze directed at oneself (e.g., Binetti et al., 2016) and on dialogical turn-taking (see introduction). Then, the initially somewhat latent receptivity on A’s side, which at first only refers to registering B’s acceptance concerning her own PF-A activity, increases to a role-inverted acceptance of such an activity, now emanating from B. We suggest that it is this change in B’s mental activity in particular that stimulates A to perform a complementary change and, although continuing to keep B physically in view, to then perceive B primarily as the mental agent expressing herself through and beyond her physical organization. In other words, while A’s attention was at first directed to B’s bodily appearance and expressive content (PF-A received by RO-B), these become transparent to A through the complementary role change for the very same mental activities of B (RO-A receiving PF-B). Consequently, nonverbal dyadic attention regulation can be reconstructed as a complementarily entangled oscillation of mental activities with the periodic sequence PF-A → RO-B → PF-B → RO-A, etc., and correspondingly alternating experiential qualities of stronger and weaker embodiment.

#### Connection to and extension of direct social perception

The preceding investigation into the sense-status of the I-Thou relation is admittedly exploratory rather than conclusive in nature. Of course, further empirical and theoretical work needs to be done to verify or falsify this hypothetical modality, but we already see some potential here to support direct social perception. What justifies locating this approach in the context of DSP is the inclusion of the two main features that set the DSP thesis apart from cognitivist accounts such as TT and ST. Here, the relational interspace between subject and object (or second subject) from an ecological psychology perspective (Gibson, 1979) and the original, observable activity of the subject (or interaction with the second subject) highlighted by enactivism (Varela et al., 1991) are to be mentioned, both of which had been neglected by cognitivism. Furthermore, besides these key aspects, our approach is non-representational and non-inferential, which is also advocated by most DSP accounts (Gallagher, 2008; Michael and de Bruin, 2015). Nevertheless, the outlined conception of an I-Thou sense deviates in some respect from other DSP approaches, the most crucial of which relates to the aspect of *embodiment* meaning that bodily expressive behavior makes up a constitutive *part of* persons’ mental states (see introduction). Although this notion is sometimes held up as a radical maxim (e.g., Chemero, 2009; Lindblom, 2020), more cautious defenders of DSP concede that not all aspects of social cognition are exhausted by either embodied or direct social cognition accounts (Krueger, 2018). This concession leaves room, from our viewpoint, for additional mental aspects and direct intersubjective, mutual observations as, for instance, the I-Thou relation. For the DSP framework, however, this requires an extension by reconsidering the “part of” relation which explains, according to the proponents of DSP, the *constitutive* role of observed embodied expressive behavior for mental phenomena. Because if something is a part of a whole and the whole cannot exist without the part, then the latter is ontologically co-constitutive of the whole (Husserl, 2001). In terms of DSP, this means that, for instance, the sad bodily posture of a person is co-constitutive of the felt (or observed) sadness (the entire emotional state or process). Indeed, we have found much evidence of this co-constitutive interrelation at the second level of analysis (e.g., under the category of Mirroring). As analysis of Level 3 has shown, however, such part-of relations do not exhaust the mental phenomena described in the data. Take, for example, Participant 19 describing a phase of eye-contact in such a way that she is proactively focused by the other person (PF-B, BRI = 0.5), feels exposed or “naked” in a metaphorical sense (PF-B, BRI = 0), and then notices the other’s “vulnerability” residing behind the proactive gesture (RO-A, BRI = 0). This encounter is initially co-constituted by the mutual gaze but then goes beyond it in the subject’s experiencing the change in the other’s mental activity to the receptive form and so coming to view the other as a mental agent on equal footing, independently of expressive bodily behavior and communicative content. This is so because eye contact functions here as a medium for the direct I-Thou encounter and recedes into the background, so to speak, by enabling the direct experience of the other’s mentally performative presence. This contrasts with the above-mentioned sad embodied behavior, which is not only a medium of the perceived sadness but at the same time a genuine part of it. But since this does not address the other person as such, but only one of her contingent mental states, we can say that for the former, eye-contact serves as a necessary but not sufficient condition in contrast to a constitutive relation, which justifies the integration of weakly embodied forms of social perception (the I-Thou relation) into an extended framework of DSP.

Besides graded forms of embodiment, this argument already includes another way in which our approach suggests an extension of DSP in terms of mental content. As pointed out above, the experience of an I-Thou encounter is not a contingent mental content, like emotions. It is rather the performative, “kinaesthetic” experience of mentally moving the other and being moved by them. Therefore, the actual content of the I-Thou sensorial experience, beyond particular mental states, is the attentional movement oscillating between the reference objects of shared attention (or intentionality), bodily appearance and mental activity, which can be conceived as a *dynamic self* (Wagemann, 2020) or *attentional self* (Watzl, 2018). Understood this way, the self as the basic unit of the I-Thou relation is not relegated to a transcendent phenomenal or even non-phenomenal transcendental realm, but rather penetrates externally perceived behavior to the point of mental affordances and can be perceived as such from both sides. Since this quasi-sensory process, in which stimulus and receptor are of a functionally complementary but phenomenally identical nature, is independent of particular mental content, it establishes another, more fundamental or, as proposed, “social-aesthetic” dimension of DSP.

Connecting this suggested extension of DSP with the aspect of unmasked vs. masked interaction may initially cause irritation: although the experimental conditions actually concern embodied perception, the effects of the study occur most strongly (in statistical terms) and in most detail (in phenomenological terms) with respect to mental activity, which relativizes embodiment to some extent. This paradox can be resolved as follows: on the one hand, mental activities decrease significantly across conditions but, on the other hand, their composition of stronger and weaker embodiment does not change. This could be taken to mean that although mask-wearing affects the quantitative occurrence of mental activities in total, it does not affect the basic, quasi-sensory mechanism mediating between more and less embodied activities (PF-A/B vs. RO-A/B). Both aspects taken together constitute the ambivalence of this mechanism in relation to embodiment: Body-related perception is necessary, on the one hand, in order to be able to interact with others at all and to perceive them as embodied persons, and, on the other hand, in order to be able to leave behind their corporeality in favor of their mental agency, which, beyond contingent communicative or pragmatic purposes, is the basic content of social perception. Social distancing through mask-wearing impairs *both aspects* of mental function related to corporeality in more and less embodied directions. While the first, strongly embodied effect of mask-wearing has already been proven for various topics (see introduction), our study reveals the other, less obvious but experientially fundamental effect on the unfolding and awareness of mental activity in interpersonal perception and corresponding interaction quality. The latter, however, seems to have been already touched upon in terms of perceived closeness (Grundmann et al., 2021; Kastendieck et al., 2022) or interpersonal trust (Malik et al., 2021; Marini et al., 2021), but only in the context of the indicated phenomenological limitations regarding the experimental setting and data collection and without the philosophical contextualization provided here.

## Conclusion and outlook

In this study, the impact of mask-wearing on social perception in nonverbal interaction was investigated in a first-person experimental design with two conditions, analyzed at three qualitative and various quantitative levels, and discussed in theoretical and philosophical regard. It is in the mixed-methods integration of these wide-ranging aspects that we see the strength of this research, which is quite close to people’s immediate experience, deploys the proven tools of empirical research, and gives a new impetus to the DSP debate by proposing a quasi-sensory modality mediating between different grades of embodiment. The crucial effect of moving from interaction without mask to interaction with mask points to characteristic, complementarily oscillating forms of mental activity that constitute this I-Thou sense and replicate previous work on social interaction (Wagemann and Weger, 2021) and other cognitive processes (Wagemann, 2020, 2022a,b).

Beyond these basic research findings and conceptions, practical implications may also arise from this study, as will be briefly indicated. For example, the structure of dyadic and monadic passages in the data can also be understood as intermittently changing engagement of participants in the sense of I and Thou. This and the varying distribution of reported mental activities across participants could raise questions about personal state or trait dispositions for using this sense. While this would of course have to be investigated in future research, it can be surmised that the I-Thou sense could probably be developed and cultivated just like other sensory modalities in educational, therapeutic, or other social (−aesthetic) contexts, which would require identifying facilitating and detrimental factors beyond the issue of mask-wearing (e.g., similar to a “Sensory Processing Sensitivity,” Lionetti et al., 2019). As Helmuth Plessner has pointed out in his “aesthesiology of the senses,” the unfolding of the discriminatory capacity of the sense modalities depends on their constant interaction with the cultural realm. To give an example, the sense of hearing develops constantly with the experience and active acquisition of musical compositions. Famously, he calls this developmental process the “spiritualization of the senses” (Plessner, 2003). How exactly this is accomplished with the I-Thou sense is a significant field of prospective research.

Furthermore, we suspect that the findings obtained here for the face mask issue would similarly emerge for other social distancing aspects such as online communication (e.g., Osler, 2020), although this would require a task accounting for the lack of direct eye contact in online settings. Particularly in the developmental context, we consider the I-Thou sense to be an important conception in that social encounters in early infancy are purely dyadic until 9 months of age, before competence in triadic relations to objects and people related to them emerges (Scaife and Bruner, 1975; Bretherton, 1991). Today, when immediate personal encounters and interactions between people of any age is becoming less and less frequent due to the digitalization of many areas of life and global pandemics, research that elucidates in equal measure the bodily, phenomenally conscious, and mentally active foundations of social encounter, and from which practical consequences can be derived, is vital.

Beyond such potential applications and continuations of this study, further research desiderata arise from its content and methodological limitations. While we have highlighted the blending of different methodological traditions as a strength, it can also be seen as a weakness in that it cannot do justice to the individual aspects in sufficient depth. From an experimental-psychological point of view, the asymmetric experimental design (without mask → with mask) and the relatively small and specific sample (in terms of age, gender, educational background) are the most important aspects that stand in the way of generalizing the findings. From a qualitative perspective, an even deeper phenomenological or hermeneutic analysis of the data would certainly be desirable, which could only be achieved here initially with a focus on mental activities. And philosophically, only the most important aspects could be addressed here – these would require further discussion within the context of related debates (especially DSP and mental action, but also cognitive phenomenology and mind–body). In these three directions, thematic and methodological focal points for further research can be added, which, however, would have no starting point without this integrative study. In this sense, we look forward to an expansion and intensified dialog in the field of transdisciplinary social cognition research.

## Data availability statement

The raw data supporting the conclusions of this article will be made available by the authors, without undue reservation.

## Ethics statement

Ethical review and approval was not required for the study on human participants in accordance with the local legislation and institutional requirements. The patients/participants provided their written informed consent to participate in this study.

## Author contributions

JW: study design and implementation, qualitative and quantitative analyses, and structure and main body of the article. CT: qualitative analysis, theoretical and philosophical aspects, methodological discussion, and article revision. JR: qualitative analysis, literature review, methodological discussion, and article revision. All authors contributed to the article and approved the submitted version.

## Conflict of interest

The authors declare that the research was conducted in the absence of any commercial or financial relationships that could be construed as a potential conflict of interest.

## Publisher’s note

All claims expressed in this article are solely those of the authors and do not necessarily represent those of their affiliated organizations, or those of the publisher, the editors and the reviewers. Any product that may be evaluated in this article, or claim that may be made by its manufacturer, is not guaranteed or endorsed by the publisher.
